# Functional gait analysis reveals insufficient hindfoot compensation for varus and valgus osteoarthritis of the knee

**DOI:** 10.1007/s00264-023-05738-5

**Published:** 2023-02-25

**Authors:** Marlene Rühling, Stephanie Kirschbaum, Carsten Perka, Frank Graef

**Affiliations:** Charité – Universitätsmedizin Berlin, corporate member of Freie Universität Berlin and Humboldt-Universität zu Berlin, Center for Musculoskeletal Surgery, Charitéplatz 1, 10117 Berlin, Germany

**Keywords:** Ankle, TKA, Knee osteoarthritis, Kinematic chain, Foot posture

## Abstract

**Purpose:**

The hindfoot is believed to compensate varus and valgus deformities of the knee by eversion and inversion movements. But these mechanisms were merely found in static radiologic measurements. The aim of this study was, therefore, to assess dynamic foot posture during gait using pressure-sensitive wireless insoles in patients with osteoarthritis of the knee and frontal knee deformities.

**Methods:**

Patients with osteoarthritis of the knee were prospectively included in this study. Patients were clinically and radiologically (mechanical tibiofemoral angle (mTFA), hindfoot alignment view angle (HAVA), and talar tilt (TT)) exa mined. Gait line analysis was conducted using pressure-sensitive digital shoe insoles.

**Results:**

Eighty-two patients (varus *n* = 52, valgus *n* = 30) were included in this prospective clinical study. Radiologically, the mTFA significantly correlated with the HAVA (*cor* = −0.72, *p* < 0.001) and with the TT (Pearson’s cor = 0.32, *p* < 0.006). Gait analysis revealed that the gait lines in varus knee osteoarthritis were lateralized, despite the hindfoot valgus. In valgus knee osteoarthritis, gait lines were medialized, although the hindfoot compensated by varization.

**Conclusions:**

Functional dynamic gait analysis could demonstrate that the hindfoot is not able to sufficiently compensate for frontal malalignments of the knee joint, contrary to static radiologic findings. This led to a narrowing of the joint space of the ankle medially in varus and laterally in valgus knee osteoarthritis.

## Introduction

Although orthopaedic surgeons are increasingly becoming sub-specialized on single joints, therapy algorithms are required to take the entire kinematic chain into account [1–3]. Mechanical corrections performed on a single joint can have vast implications for neighbouring joints, as well [4]. Recently, clinical studies reported that following total knee arthroplasty (TKA), patients may develop ankle symptoms, particularly if patients had an excessive pre-operative varus or valgus malalignment [5–8]. It is necessary to identify the mechanisms of the ankle and knee joint mutually affecting each other—both pre-operatively in knee osteoarthritis and following TKA. This would enable clinicians to improve conservative treatment of knee osteoarthritis by additionally taking care of the ankle joint. Secondly, the onset or progression of ankle symptoms following TKA could be prevented.

The biomechanical implications of altered kinematics in osteoarthritic knees on the ankle joint can be manifold and include rotational, frontal, and sagittal changes. Conversely, compensatory changes at the ankle joint, such as an altered rotation, could influence, e.g., tibia rotation and subsequently patellofemoral function [9–11]. Recent studies claimed that the frontal malalignment of varus or valgus knee osteoarthritis can be compensated by the hindfoot [12, 13]. In varus knee osteoarthritis, the hindfoot compensates by eversion, i.e., by going into valgus. And in valgus knee osteoarthritis, the hindfoot compensates by inversion, i.e., by going into varus. But these studies could not sufficiently explain why patients developed ankle pain after TKA.

The main issue of these studies was that they merely analyzed static radiologic parameters retrospectively and did not take dynamic functional foot and ankle posture parameters into account. The interplay between the ankle, hindfoot, and forefoot during gait is a complex orchestrated process. In short, in the first phase of the gait cycle, the “heel strike,” the subtalar joint is moved into valgus, which is discussed to unlock the midtarsal joints so that they become less stiff and can act as a shock absorber to the body weight. In the last phase of the gait cycle, the “toe off phase,” the subtalar joint moves into varus, which locks the midtarsal joints to become a rigid lever during push off [14, 15]. As a consequence, measurements of the ankle and subtalar joint done on static X-rays cannot sufficiently account for these dynamic movements. And therefore, retrospective radiologic studies on the interplay between the knee and ankle joint are unable to explain why patients developed ankle pain following TKA, specifically, when discussing changes of the frontal mechanical axis of the knee and ankle joint.

In order to assess those dynamic movements, it is essential to record load distribution while walking. Digital pressure sensitive insoles can be worn just as regular insoles in shoes and are able to capture acceleration and pressure data during gait. Insoles of certain manufacturers have been shown to be reliable in measuring gait parameters such as the vertical ground reaction force or the center of pressure, allowing the examiner to analyze specific pressure distribution changes, such as in the medio-lateral direction [16, 17]. Compared to conventional pedography using a fixed platform, on which patients put a single step for the analysis, digital pressure insoles allow to record multiple gait cycles in a row and calculate mean values [18].

The aim of the present study was to analyze medio-lateral pressure distribution changes of different phases of the gait cycle in patients with osteoarthritis of the knee using digital pressure insoles. The main hypothesis of this study was that the degree of varus deformity at the knee joint correlated with a medialization of the gait line, because radiologically, the hindfoot shifts into eversion. And that the degree of valgus deformity at the knee joint correlated with a lateralization of the gait line, corresponding to the inversion of the hindfoot seen radiologically.

## Methods

This study was approved by the local ethics committee (approval number: AS 116(bB)/2019). Written informed consent was obtained from all patients. The study protocol was registered at the German Clinical Trials Register (DRKS-ID: DRKS00017400).

### Patient selection

From September 2020 until September 2021, patients with osteoarthritis of the knee were included in this study. The study was conducted at a German university hospital. For this clinical prospective level II study, the STROBE guidelines for reporting observational studies were followed [19]. Inclusion criteria were: end-stage osteoarthritis of the knee, all genders, age > 18 years, and willingness to participate. Exclusion criteria were: rheumatoid arthritis, previous hindfoot operations or joint fusions of the foot and ankle, post-traumatic pathologies/osteoarthritis of the foot and ankle joint, neurologic disorders or polyneuropathy affecting gait and postural control (e.g. Parkinson’s disease), progressed diabetes, and Charcot’s foot.

### Clinical examination

Patients were clinically examined by measuring the ROM in extension and flexion of the knee and ankle joint. The ROM of the ankle joint was measured with the knee in 90° flexion. For statistical analysis, if a motion deficit of, e.g., extension/flexion 0-5-90° was present, the extension deficit was documented as extension = −5°.

### Radiologic analysis

Full weight-bearing anteroposterior (ap) X-rays of the whole lower limb were acquired with the leg in neutral rotation, the patella facing straight forward, the fibular head covered by the tibia for one third, and correct projection of the trochanter minor and ankle, to minimize measurement inaccuracies caused by internal or external lower leg malrotation in varus or valgus osteoarthritis [20–22]. Lateral radiographs of the standing knee were also taken. When patients demonstrated a varus or valgus malalignment of the whole lower limb ≥ 5°, additional standing full weight-bearing X-rays of the foot and ankle joint were acquired: an ap mortise view of the ankle joint, a lateral view of the foot, and ankle and a hindfoot view [23].

The mTFA was defined as the angle between the femoral and tibial mechanical axis [24]. The HAVA was defined as the angle between the mechanical tibial axis and a line running from the most distal tip of the calcaneus to the intersection of the tibial axis with the ankle joint line in the hindfoot view [13, 25]. Positive values corresponded to varus and negative values to valgus alignment.

The Meary’s angle was measured in lateral standing foot x-rays to evaluate if a flat foot or cavus deformity was present. The Meary's angle is defined as the angle between the midline axis of the talus and the axis of the first metatarsal [26]. Positive values were defined for Pes planus deformities, negative values for cavus deformities. The talar tilt (TT) was defined as the angle of the intersection between the horizontal tangent of the tibial plafond and the horizontal tangent of the talus dome [27]. Positive values corresponded to varus and negative values to valgus alignment.

### Gait analysis

Gait analysis was performed using wireless pressure-sensitive insoles (Moticon ReGo AG, Munich, Germany, Insole Model 3) and a 100 Hz sampling rate. For each patient, an individual insole size was chosen according to their shoe size. Prior to each measurement, patients wore the sensor insoles for six minutes and were asked to walk 20 steps to allow for acclimatization and warmup of the sensor insoles [28]. Patient data was recorded while patients were walking a 20 metre straight line on even ground.

Gait parameters were analyzed using the OpenGo software (Moticon ReGo AG, Munich, Germany). Here, the vertical ground reaction force (vGRF) was measured in Newton (N). These values were then manually normalized by division by the body weight in kilogram (BWkg) and the gravity of earth (g) to allow for a dimensionless inter-individual comparability [29]. Gait lines were calculated using the software based on centre of pressure (COP) values. The software in combination with the insoles has an algorithm to automatically detect steps and verify measurements as eligible for gait analysis.

**Fig. 1:**
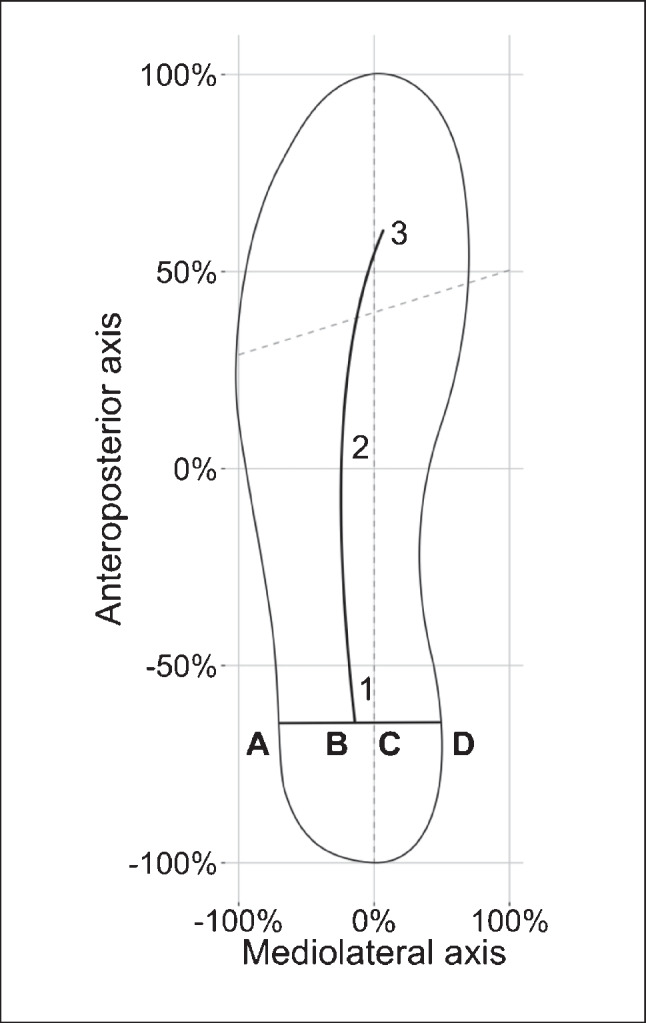
The MLEI was determined for three points. (**A**) The beginning of the gait line (MLEI 1), corresponding to the heel strike or initial contact. (**B**) The intersection between the gait line and the mediolateral axis corresponding to the mid-stance (MLEI 2). (**C**) The end of the gait line, corresponding to the toe off phase (MLEI 3). The MLEI was then calculated by dividing the distance **BC** by the width of the sole at the specific location (distance **AD**) multiplied by 100 (*MLEI* = *BC*/*AD* × 100)

**Fig. 2:**
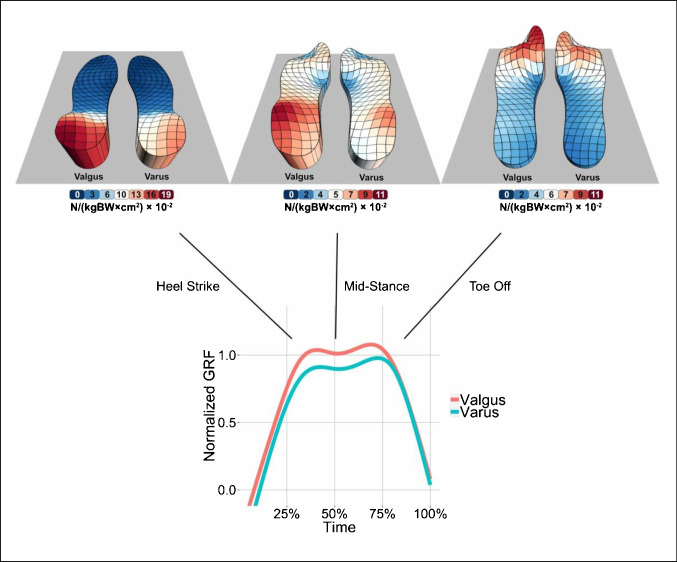
Mean pressure distribution 3D visualization of all valgus and varus patients for the three time points of the gait cycle (heel strike, mid-stance and toe off). Valgus patients had higher pressure values at the medial portion of the insoles compared to varus patients, who had higher pressure values laterally. The ground reaction force (GRF) was normalized by division by the body weight in kg (kgBW) and the gravity of earth (g)

To quantify the degree of medio-lateral deviation of the gait line, the medio-lateral excursion index was defined for three positions (MLEI 1-3), an adjusted method of the centure excursion pressure index [30]. The MLEI was calculated by dividing the distance from the gait line to the longitudinal bisection of the insole (anteroposterior axis) and the width of the insole at that position (Fig. 1). These positions were chosen based on the three phases of gait “heel strike,” “mid-stance,” and “toe off” (Fig. 2) [31]. Lower MLEI values correspond to more lateral, higher MLEI values to more medial gait line excursions.

### Statistical analysis

Statistics were performed using “R” and the software RStudio© (RStudio, Inc., Boston, USA). Sample size calculation was conducted using the “pwr” package. For a given medium effect size and a power of 80%, *n* = 82, patients were required for answering the main hypothesis. Data were analyzed concerning normal/nonnormal distribution using histograms, QQ-plots, mean/median, and skewness. Correlations were displayed with scatter plots and calculated using Pearson’s (continuous data) or Spearman’s (ordinal data) correlation coefficient. Differences between two independent groups with nonnormal distribution were calculated using two-sided Wilcoxon signed rank tests. Independent categorical variables were tested using the Exact Fisher’s test. The significance level was *p* < 0.05. The Bonferroni correction was applied for multiple comparisons.

## Results

Eighty-seven patients were both willing to participate in this study and were deemed eligible for study inclusion. Five patients were excluded, leaving a total of 82 patients (varus *n* = 52, valgus *n* = 30) for study inclusion. Patient baseline characteristics are displayed in Table 1.

**Table 1:** Baseline characteristics

	Valgus	Varus	*p*
*n*	30	52	
BMI (median [IQR])	25.84 [23.28, 30.39]	30.31 [21.07, 35.41]	0.004
Sex (%)			
m	5 (16.7)	26 (50.0)	0.004
w	25 (83.3)	26 (50.0)	
Side (%)			
Left	9 (30.0)	23 (44.2)	0.245
Right	21 (70.0)	29 (55.8)	

Wilcoxon signed rank test for non-parametric data and Exact Fisher’s test for categorical variable. The significance level was *p* < 0.05

*mTFA*, mechanical tibiofemoral angle

**Fig. 3:**
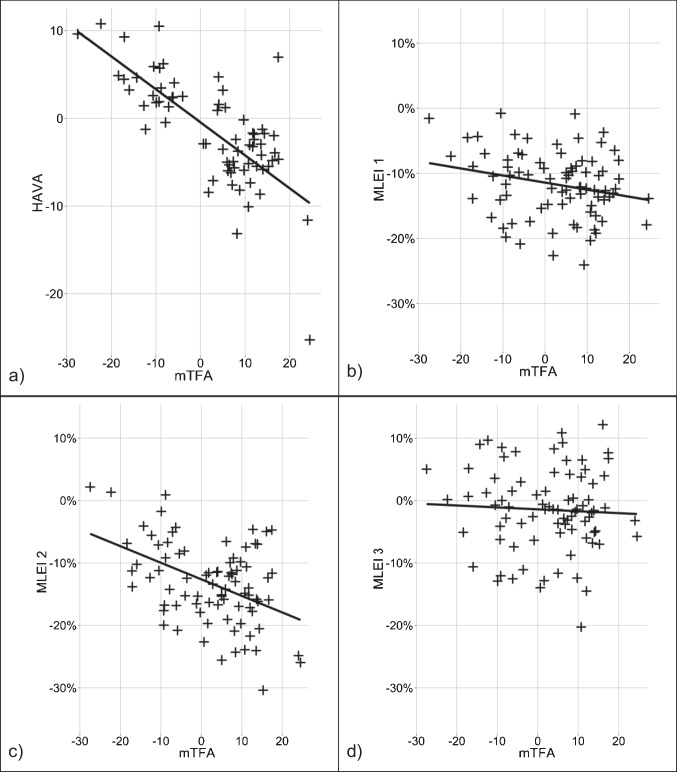
Correlation analysis between **a** mTFA and HAVA showed a strong negative correlation (*cor* = -0.72, *p* < 0.001). **b** No significant correlation between MLEI 1 and mTFA (*rho* = −0.19, *p* = 0.078), and **d** MLEI 3 and mTFA (*rho* = −0.065, *p* = 0.56) could be demonstrated. A significant correlation between **c** MLEI 2 and mTFA was shown (*rho* = −0.33, *p* = 0.002)

Radiologic analysis demonstrated a strong significant correlation between the mTFA and HAVA measurements (*cor* = −0.72, *p* < 0.001). Higher grades of varus malalignment at the knee joint were associated with higher grades of valgus at the hindfoot. Higher grades of valgus malalignment at the knee joint were associated with higher grades of varus at the hindfoot, radiologically (Fig. 3).

**Fig. 4:**
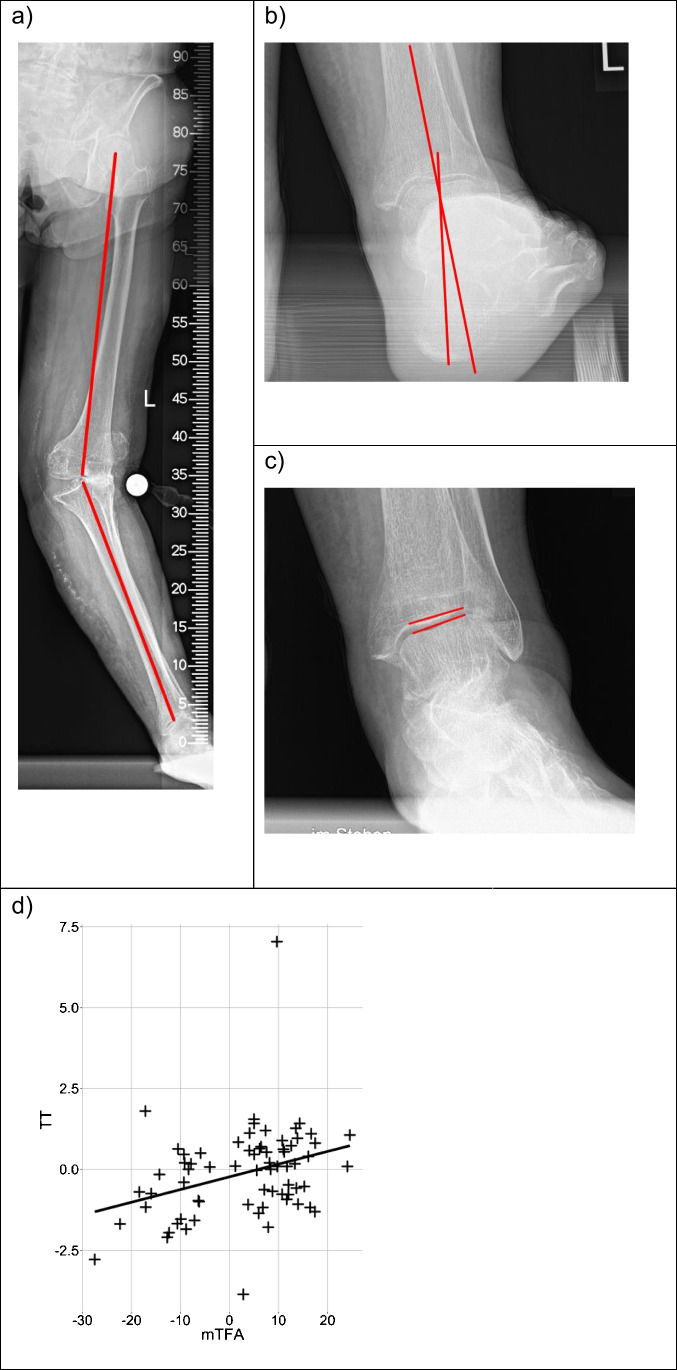
Case of a 93 year-old female patient with valgus osteoarthritis of the knee and a mTFA of −27.5° (**a**). The hindfoot compensated by varization of 9.6° (**b**). Nonetheless, a negative TT of −2.7 was seen, indicating that the compensation mechanisms of the subtalar joint were insufficient (**c**). A significant correlation was found between the mTFA and TT (Pearson’s cor = 0.32, *p* < 0.006) (**d**), indicating that higher grades of valgus at the knee joint were associated with higher grades of valgus malalignment at the ankle joint and vice versa. The automatic step-detection algorithm of the software and the sensor insoles verified the data of this patient as eligible for gait analysis

The Meary angle for measuring cavus or planus deformities of the foot was 8.77° (± 7.84° SD) in valgus and 8.21° (± 7.23° SD) in varus patients. These mean that Meary’s angles were physiological and did not differ statistically between both groups [32]. Evaluation of the alignment of the ankle joints demonstrated that the TT was significantly different between both groups. In the valgus group, the TT was oriented in valgus. And in the varus group, the TT was oriented in varus (Table 2). Albeit the TT was significantly different between both groups, it was not pathological for neither of one of the groups [33]. Correlation analysis revealed that the TT significantly correlated with the mTFA (Pearson’s cor = 0.32, *p* < 0.006) indicating that higher grades of valgus at the knee joint were associated with higher grades of valgus malalignment at the ankle joint and vice versa (Fig. 4). Figure 4 presents the case of a 93 year-old female patient with an excessive valgus osteoarthritis of the knee (mTFA of −27.5°). The hindfoot compensated by varization of 9.6°. The TT was measured −2.7°, corresponding to a moderately valgus alignment of the ankle joint.

**Table 2:** Clinical and radiologic measurements

	Valgus	Varus	*p*
*n*	30	52	
ROM ankle dorsiflexion (median [IQR])	5.00 [0.75, 10.00]	5.00 [0.00, 10.00]	0.992
ROM ankle plantarflexion (median [IQR])	35.00 [30.00, 40.00]	35.00 [30.00, 40.00]	0.707
ROM knee extension (median [IQR])	−10.00 [−13.75, 0.00]	−5.00 [−10.00, 0.00]	0.053
ROM knee flexion (median [IQR])	110.00 [90.00, 118.75]	115.00 [103.75, 120.00]	0.060
MLEI			
MLEI 1 (median [IQR])	−9.52 [−14.02, −7.00]	−12.45 [−14.81, −9.68]	0.070
MLEI 2 (median [IQR])	−10.73 [−15.31, −5.86]	−14.99 [−19.23, −11.47]	0.004
MLEI 3 (median [IQR])	−0.36 [−5.96, 3.42]	−1.52 [−4.92, 2.97]	0.651
Meary‘s angle (median [IQR])	7.62 [3.38, 14.09]	7.56 [3.80, 12.09]	0.766
TT (median [IQR])	−0.75 [−1.63, 0.12]	0.18 [−0.67, 0.83]	0.003

*MLEI*, mediolateral excursion index; *ROM*, range of motion; *TT*, talar tilt

The significance level was *p* < 0.05. Negative ROM values correspond to a motion deficit

Results of the clinical examination showed that both knee extension and flexion were significantly reduced in patients with valgus compared to varus knee osteoarthritis. There were no statistical differences in ankle ROM (Table 2).

**Fig. 5:**
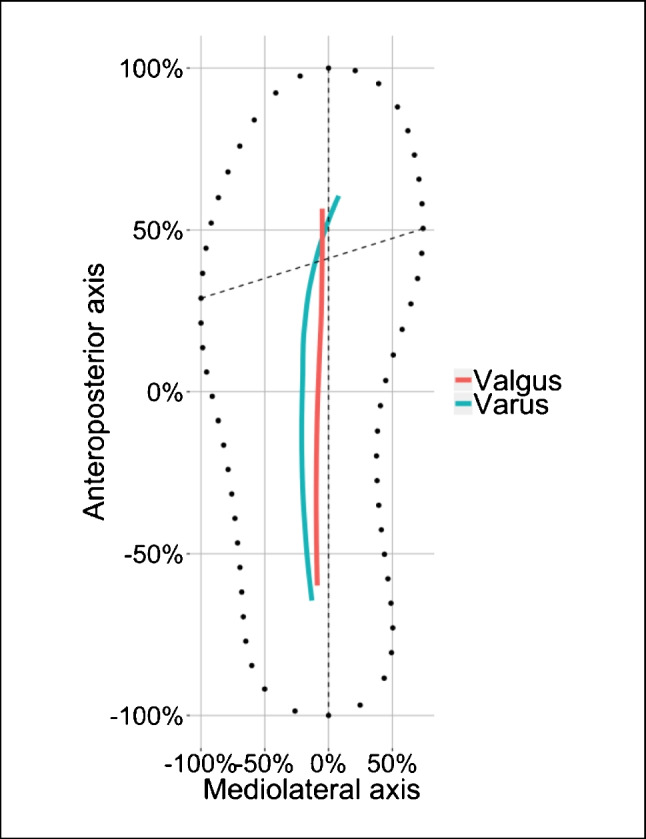
Mean gait lines of all patients grouped by valgus or varus malalignment at the knee joint. The vertical dashed line displays the ap-axis (longitudinal bisection of the sole); the horizontal dashed line displays the metatarsal axis

Contrary to the radiologic measurements, results of the MLEI gait analysis showed that at all three measurement points, higher grades of varus malalignment at the knee joint were associated with more lateral MLEI values. And higher grades of valgus malalignment at the knee joint were associated with more medial MLEI values (Fig. 5). These correlations were significant at measurement point 2. Subsequently, patients with varus osteoarthritis of the knee demonstrated more lateral gait lines, and in valgus knee osteoarthritis, the mean gait line was more medial (Fig. 5, Table 2).

The fact that knee varus osteoarthritis was associated with a lateralization of the gait line and knee valgus osteoarthritis with a medialization could also be demonstrated by a qualitative 3D pressure distribution analysis for the three time points of the gait cycle (Fig. 2).

## Discussion

The main reason to conduct this study was to analyze if the compensatory mechanisms of the hindfoot for frontal knee deformities, as already reported in static radiologic measurements, translated into functional results using dynamic gait analysis. The most important and new finding of this study was that patients with varus osteoarthritis of the knee demonstrated a lateralization of the gait line, despite radiological valgization of the hindfoot. Vice versa, patients with valgus osteoarthritis of the knee showed a medialization of the gait lines, despite radiological varization of the hindfoot.

Therefore, this study could demonstrate that the hindfoot was not able to sufficiently compensate for the changes of medio-lateral foot pressure distributions induced by high-grade frontal malalignments of the knee joint.

The valgization and varization of the subtalar joint to compensate frontal knee deformations can potentially influence the biomechanical statics of the entire foot. Hindfoot varus can lead to pes cavus deformities and hindfoot valgus to pes planus deformities [12, 13, 34, 35]. Therefore, a varus deformity at the knee joint can lead to a hindfoot valgus and consequently to a pes planus deformity, for instance. It has been reported that, using clinical tests such as the navicular height or measuring the arch index by static foot prints, patients with varus knee osteoarthritis exhibit a more pronated or flat foot [36]. Flat foot deformities can lead to a medialization of the COP in gait analysis and cavus deformities to more lateral COP values [37]. In the present study, planus or cavus deformities were measured radiologically using Meary’s angle. But no significant differences could be found between both groups in this study cohort, and in both groups, the mean Meary angle was physiological. Moreover, gait lines in knee varus patients were demonstrated to be lateralized despite the valgization of the hindfoot and vice versa.

The role of the subtalar joint in compensating for frontal malalignments of the knee joint is discussed to be crucial [27, 38]. Reasons for that are biomechanical cadaver studies, which could show that varus/valgus malalignments of the knee joint led to significantly altered contact pressure distributions in the ankle joint, especially when the ROM of the subtalar joint was fixed [39, 40]. In case of a decreased ROM of the subtalar joint, compensation of valgus/varus malalignments in the knee joint might then take place in the ankle joint and lead to ankle osteoarthritis [27, 38]. A recent study, however, found that in patients with osteoarthritis of the knee joint, higher degrees of varus or valgus deformation at the knee joint were not associated with a decrease or increase of the ROM of the subtalar joint [41]. Clinically measuring the ROM of the subtalar joint is inaccurate because the ROM of the subtalar joint is a combination of the movement of the joint itself and the stability of the medial and lateral ligaments of the ankle joint. The study therefore also concluded that frontal knee deformities do not attenuate ankle ligaments and do not provoke ankle instabilities.

Other studies reported on increased pain and impaired function of the ankle joint following TKA, particularly in high-grade varus or valgus knee osteoarthritis when the mechanical axis was corrected beyond > 10–15° [5–8]. But these studies could not clarify whether TKA leads to the onset or progression of ankle pain or if the ankle pain had already been present but was masked by symptoms of knee osteoarthritis. Tallroth et al. reported in their study of patients undergoing TKA that almost 30% of the study cohort presented with a coexisting osteoarthritis in the ankle joint, and higher grades of preoperative malalignment were associated with worse osteoarthritis grades in the ankle joint [42].

Similarly, it has been reported that organ donors who showed degenerative changes in the ankle joint also had cartilage defects in the knee joint [43]. It was therefore suggested that degenerative changes in the ankle joint can influence the knee joint, too. It needs to be discussed if the hindfoot deformation (the radiological valgus and varus position as seen in this study) was the primary cause of the limb axis deviation and the knee frontal plane deformity a consequence thereof. In another study on the relationship between cartilage wear of the knee and ankle joint using organ donors, it was found that if the knee joints of the donors had a high-grade cartilage degeneration, the ankle joints had degenerative changes, too. But the reverse was never the case [44].

In the present study, the ankle alignment was also evaluated radiologically to screen for signs of osteoarthritis. Contrary to Tallroth’s study, the mean TT was physiologic in both groups [33]. Nevertheless, measurements of the TT indicated that in valgus patients, the TT was oriented in valgus and in varus patients, and the TT was oriented in varus. And this association was found to be significantly correlated. These observations support the findings of the gait analysis, because due to an insufficient compensation mechanism in the subtalar joint, knee varus and valgus led to an increase of lateral or medial joint narrowing in the ankle joint.

These results should be taken into account when treating patients with varus or valgus osteoarthritis of the knee. In varus osteoarthritis, insoles with a lateral arch support could help to medialize the gait line. And in valgus osteoarthritis, insoles with a medial arch support could help to lateralize the gait line.

## Conclusions

High-grade frontal malalignments of the knee joint cannot be sufficiently compensated by the hindfoot. These results should be taken into account when treating patients with varus or valgus osteoarthritis of the knee by, e.g., using insoles with a medial or lateral arch support.

## Data Availability

Data are not available in a public repository.
